# Stiff limb syndrome: a case report

**DOI:** 10.1186/1757-1626-3-60

**Published:** 2010-02-12

**Authors:** Abderrazak Hajjioui, Karima Benbouazza, Mohamed El Alaoui Faris, Amina Missaoui, Najia Hajjaj Hassouni

**Affiliations:** 1 Physical Medicine and Rehabilitation, El Ayachi Hospital, Mohamed V University, Coast Road Street, Sale, 11000, Morocco; 2 Rheumatology, El Ayachi Hospital, Mohamed V University, Coast Road Street, Sale, 11000, Morocco; 3 Neurology, Cheikh Zaid Teaching Hospital, Mohamed V University, Hay Riad Street, Rabat, 10000, Morocco; 4 Internal medicine, Cheikh Zaid Teaching Hospital, Mohamed V University, Hay Riad Street, Rabat, 10000, Morocco; 5 Rheumatology, El Ayachi Hospital, Mohamed V University, Coast Road Street, Rabat, 10000, Morocco

## Abstract

**Introduction:**

Stiff limb syndrome is a clinical feature of the stiff person syndrome, which is a rare and disabling neurologic disorder characterized by muscle rigidity and episodic spasms that involve axial and limb musculature. It is an autoimmune disorder resulting in a malfunction of aminobutyric acid mediated inhibitory networks in the central nervous system. We describe a patient diagnosed by neurological symptoms of stiff limb syndrome with a good outcome after treatment, and a review of the related literature.

**Case presentation:**

A 49-year-old male patient presented with a progressive stiffness and painful spasms of his both legs resulting in a difficulty of standing up and walking. The diagnosis of stiff limb syndrome was supported by the dramatically positive response to treatment using diazepam 25 mg/day and baclofen 30 mg/day.

**Conclusion:**

This clinical case highlights the importance of a therapeutic test to confirm the diagnosis of stiff limb syndrome especially when there is a high clinical suspicion with unremarkable electromyography

## Introduction

Stiff person syndrome (SPS) is characterized by axial rigidity, progressive stiffness, and spontaneous, reflex- or actioninduced painful spasms of the paraspinal, abdominal and occasionally proximal leg muscles associated with a lumbar hyperlordosis [1]. Electrophysiological findings are typically continous motor unit activity with abnormal exteroceptive reflexes, but a normal interference pattern during spasms [2].

Most patients have antiglutamic acid decarbolxylase (GAD) antibodies in both serum and cerebral spinal fluid (CSF) with additional evidence of autoimmune disease. During the last few years, several cases with SPS were reported whose symptoms were confined to one lower limb [3]. the condition was named as the "stiff leg" or "stiff limb" syndrome (SLS). We describe a patient with signs and symptoms closely resembling those seen in cases of SLS.

## Case presentation

A 49-year-old man from a North African origin and with a Moroccan nationality presented with a 2-year history of progressive stiffness and painful spasms of his both legs, with recent worsening of his condition over the last few weeks resulting in a considerable difficulty of standing up and walking. There was no past history of diabetes, or other auto-immune diseases. The family history was unremarkable. On admission, the lower limbs were rigid with a flexum of the hips and knees, movements were severely limited and painful, and strength could not be assessed because of rigidity and spasms. A slightly increased tone was noted in his upper limbs, with normal muscular strength and without any movement limitation. No paraspinal or axial contractions were palpated. Sensory examination was normal except for reduced vibration sense in both lower extremities. Deep tendon reflexes were normal in upper limbs. Patellar and Achilles jerks could be elicited markedly. Plantar response was flexor bilaterally. Functionally the patient was unable to walk because of the rigidity in his both legs. He had an intact intellect and there were no other neurologic abnormalities.

Results of routine laboratory tests including full blood count (FBC), serum electrolytes, blood urea nitrogen, creatinine, glucose and liver enzymes were normal. The erythrocyte sedimentation rate (ESR) and C-reactive protein (CRP) values were normal. Chest X-rays and abdominal ultrasonography revealed no abnormalities. Magnetic resonance imaging of the whole neuraxis and EEG were also normal. Cerebrospinal fluid examination was unremarkable, there were no atypical cells found on cytopathological analysis. Electromyography of the lower limbs revealed no abnormalities. Serologic testing for HIV, syphilis, viral hepatitis B and C was negative. Anti-mitochondrial antibodies and anti-cytoplasm antibodies were found in the serum, while antinuclear antibodies (ANA) and anti- DNA screening and auto-antibodies to glutamic acid decarboxylase (GAD) were within normal limits. Anti-amphiphysin antibodies test wasn't carried out. With the high suspicion that the clinical case could be a SLS, oral diazepam was administered in high doses (25 mg/day) after which we observed a dramatic remission of the clinical state with marked reduction of the hypertonus and the patient could walk with little aid. The administration of 30 mg of baclofen daily provided relief of symptoms.

## Discussion

Our patient's symptoms had an insidious onset and chronic progressive course. The stiffness and painful spasms were quite confined to the lower limbs, the axial muscles being spared. Spasms induced by movement or other stimuli were an important cause of disability. Brainstem and pyramidal sings were absent and muscle strength seemed to be normal except when interrupted by spasms or hampered by severe rigidity. Extensive diagnostic work-up including imaging of the whole neuraxis and search for evidence of an infectious, malignant, or inflammatory cause did not reveal another etiology. Other potential causes of similar symptoms such as neuromyotonia were also ruled out by neuroimaging and electrophysiological studies. Even if Immunocytochemistry studies failed to demonstrate anti-GAD auto-antibodies, our patient's history, clinical examination findings, and response to benzodiazepines were all very suggestive of SLS.

"SLS" is a variant of "stiff person syndrome" (SPS) which is a rare auto-immune neurological condition first described in 1956 by Moersch and Woltman [2]. It is characterized by progressive muscular rigidity and painful spasm.

SPS remains a clinical diagnosis with the following criteria: [1] A prodrome of episodic aching stiffness of axial muscles [2]. Progression to include stiffness of proximal limbs [3]. Painful spasms elicited by triggers [4]. Increased lumbar lordosis (5) Normal sensation, motor function and intellect. (6) Electromyographic findings typical of continuous motor activity that can be abolished by diazepam administration [3].

Abnormal cutaneomuscular reflexes argue against the diagnosis of SLS and should prompt the search for another cause [4].

The cause of stiff-person syndrome is unknown but an auto-immune pathogenesis is suspected because of the presence of antibodies against glutamic acid decaroxylase (GAD), the rate-limiting enzyme for synthesis of the inhibitory neurotransmitter gamma-aminobutyric acid (GABA) [1,5], and the association with other auto-immune diseases such as diabetes and thyroiditis [6].

The loss of GABAergic input into motor neurons is thought to produce the tonic firing of motor neurons at rest and lead to their excessive excitation in response to sensory stimulation [1]. Patients with SPS have high titter GAD-antibodies which are synthesized intra thecally and seem to impair the in situ synthesis of GABA [6].

SLS is a newly emerging entity considered as a focal form of SPS in which the symptoms are confined to a distal limb (usually the leg), although sometimes this progresses to involve the axial musculature as well [4].

In this group of patients, sphincter disturbance may develop years after the onset; abnormally segmented electromyographic (EMG) activity is recorded during spasms in addition to the electrophysiological findings of SPS; anti-GAD antibodies and autoimmune diseases are less common, [7] as in our case. The standard therapy for patients has been the GABA neuromodulator diazepam. Initial results are usually good, which was the case of our patient, but adaptation and disease progression make increased dose up to 200 mg daily necessary. The subsequent side effects as well as the risk of addiction limit the applicability of high-dose oral therapy [7].

Improvement of SPS symptoms with immunotherapy provides additional strong support for the auto-immune hypothesis of the disease. Plasmapheresis has had variable effect [8]. Intravenous immunoglobulin therapy has been more successful [9]. Physical therapy can substantially improve quality of life [10].

## Consent

Written informed consent was obtained from the patient for open access publication of this case report.

## Competing interests

The authors declare that they have no competing interests.

## Authors' contributions

All authors have made significant contributions by making diagnosis and intellectual input in the case and writing the manuscript. All authors read and approved the final manuscript.
